# A prognostic index for locoregional recurrence after neoadjuvant chemotherapy

**DOI:** 10.3332/ecancer.2016.647

**Published:** 2016-06-16

**Authors:** C Herrero-Vicent, A Guerrero-Zotano, J Gavilá-Gregori, A Hernández-Blanquisett, S Sandiego-Contreras, JM Samper-Hiraldo, V Guillem-Porta, A Ruiz-Simón

**Affiliations:** Medical Oncology, Valencian Institute of Oncology, Valencia, Spain

**Keywords:** prognostic index, locoregional recurrence, breast cancer

## Abstract

**Background:**

The appropriate selection criteria for breast-conserving surgery (BCS) or mastectomy after neoadjuvant chemotherapy (NAC) are poorly defined. The aim of this study is to analyse the incidence and prognostic factors for locoregional recurrence (LRR) in patients with breast cancer (BC) treated with NAC to develop a prognostic score to help with clinical decision-making.

**Materials and methods:**

Using our retrospective maintained BC database, we identified 730 patients treated with NAC (327 patients treated with BCS and 403 patients treated with mastectomy) between 1998 and 2014. To identify variables associated with an increased LRR rate, we performed firstly Kaplan–Meier curves, with comparisons among groups using log-rank test, and then, significant variables were included in a multivariate analysis using Cox proportional hazards. The prognostic index was developed by assigning score 0 (favourable) or score 1 (unfavourable) for each significant variable of multivariate analysis and was created separately for patients with BCS and mastectomy.

**Results:**

At a median follow-up of 72 months, the 6-year cumulative incidence of LRR was 7.2% ( ± 3%) for BCS and 7.9% ( ± 3%) for mastectomy.

By univariate analysis, variables associated with an increased LRR were for BCS: HER2 positive, grade III, ductal carcinoma *in situ* (DCIS), No-pCR (ypTis, ypN0), and age < 40 years; and for mastectomy, HER2-positive, DCIS, No-pCR, and LVI. By multivariate analysis, variables associated with an increased LRR were for BCS: HER2 positive (HR: 11.1, *p* = 0.001), DCIS (HR: 3.1, *p* = 0.005), and age < 40 years (HR: 2.8, *p* = 0.02); and for mastectomy: HER2 positive (HR: 9.5, *p* = 0.03), DCIS (HR: 2.7, *p* = 0.01), No-pCR (HR: 11.4, *p* = 0.01), and age < 40 years (HR: 2.8, *p* = 0.006).

The score stratified patients into three subsets with statistically different levels of risk for LRR. For BCS, the six-year LRR rates were 3%, 13%, and 33% for the low (score 0, *n* = 120), intermediate (score 1, *n* = 95) and high (score 2–3, *n* = 27) risk groups, respectively (*p* = 0.001). For mastectomy, the six-year LRR rates were 0%, 8%, and 27% for the low (score 0, *n* = 20), intermediate (score 1–2, *n* 191), and high (score 3–4, *n* = 30) risk groups, respectively (*p* = 0.001). Of note, 21 patients that had a LRR event were HER2 positive, all of them had received trastuzumab.

**Conclusions:**

Patients with a score of 0, which made up to 19% of the study population, had very low risk of LRR. The score enabled the identification of a small group (7%) of patients with very high risk of LRR, and who may benefit from alternative treatment.

## Introduction

Currently, neoadjuvant chemotherapy (NAC) is widely accepted in the management of operable and inoperable breast cancer tumours [1–3]. The benefits of this approach include permitting the *in vivo* assessment of disease response to a particular chemotherapy schedule, and allowing selected patients in whom mastectomy was recommended initially the opportunity to undergo breast-conserving surgery (BCS) [4–9].

Nevertheless, there is a concern that the rates of locoregional recurrence (LRR) may be higher than those reported when surgery is used first. Some studies have reported LRR rates ≤ 30% of patients treated with NAC and surgery [10–15]. The most important prognostic factors in early breast cancer for patients who receive surgery as their initial treatment are oestrogen receptors, progesterone receptors, proliferation markers, number of involved lymph nodes, tumour histology, size, grade, and the presence of peritumoural vascular invasion, and additionally, in breast conservative surgery, the ipsilateral breast recurrence risk related to the status of surgical margins and the presence of extensive intraductal components. Meanwhile, human epidermal growth factor receptor 2 (HER2) overexpression is both a prognostic and a predictive factor. However, there is limited information on rates and prognostic factors of LRR for patients who receive neoadjuvant chemotherapy [16–19].

Currently, the appropriate selection criteria for BCS or mastectomy after neoadjuvant chemotherapy are poorly defined.

The aim of this study is to analyse retrospectively incidence and prognostic factors for locoregional recurrence in breast cancer patients treated with NAC to develop a prognostic score to help for clinical decision-making.

## Materials and methods

The data from 730 consecutive patients with histologically confirmed breast carcinoma treated with NAC (327 patients treated with breast conservative-surgery and 403 patients treated with mastectomy) between 1998 and 2014 at the Valencian Institute of Oncology. Demographical, clinicopathological, and treatment variables were abstracted retrospectively from the data and medical histories of each patient.

At presentation, disease status was assessed using diagnostic assessments such as medical history, including family cancer history and menopausal status, a physical examination including bimanual palpation of the breasts and locoregional lymph nodes, radiological examination including bilateral mammography and ultrasounds of the breast and regional lymph nodes. Moreover, all patients underwent a laboratory assessment including full blood account, liver, and renal function tests, alkaline phosphatase and Ca 15.3, chest–abdominal and pelvic computed tomography or abdominal ultrasound, and bone scintigraphy in order to exclude the presence of metastatic disease. Although breast cancer magnetic resonance imaging (MRI) is the most accurate modality for assessing the extent of residual disease following NAC, not all patients of the study underwent a breast cancer MRI.

Patients were pathologically staged in accordance with the World Health Organisation (WHO) classification (4th edition) and the American Joint Committee on Cancer (AJCC)/Union for International Cancer Control (UICC) tumour nodes metastasis (TMN) staging classification system (7th edition).

All patients were diagnosed by core needle biopsy using ultrasounds, or using bimanual palpation, providing information on histological type and grade, oestrogen receptor (ER), progesterone receptor (PgR) and from 2005 also HER2 status. ER and PgR status was assessed by immunohistochemistry (IHC), and HER2 status was assessed by either fluorescent *in situ* hybridisation (FISH) or validated IHC method (Herceptest). For ER and PgR, cases were considered as negative when the percentage of immunoreactive tumour cells was below 1%, and the remaining cases > 1% of tumour cells stained were classified as positive. For Her2, cases were considered positive if Herceptest results 3+ and/or FISH showed a ratio Her2/CEpT < 2 and the remaining cases were classified as negative. For the purpose of treatment decision-making, tumours were grouped into surrogate intrinsic subtypes defined by routine histology and immunochemistry following the intrinsic subtypes of breast cancer of St Gallen Conference 2013.

Preoperative NAC included both anthracyclines and taxanes. The most frequently used regimen included cyclofosphamide 600 mg/m^2^/21 days and doxorubicin 60 mg/m^2^/21 days for four cycles, followed by docetaxel 100 mg/m^2^/21 days for four cycles. After its approval, trastuzumab was administered concomitantly with taxanes to patients whose tumours overexpressed Her2. After surgery, hormone therapy was administered in all tumours with positive hormone receptors, and after approval adjuvant trastuzumab was given to patients whose tumours overexpressed Her2.

Before and after NAC, all patients were evaluated by a multidisciplinary team, including at least a surgeon, radiation oncologist, medical oncologist, radiologist and pathologist, all of whom specialised in breast cancer. The team determined eligibility for BCS or mastectomy depending on the reduction of the primary tumour with neoadjuvant therapy. In the breast-conservative surgery group of patients, when final pathological examination indicated positive margins, patients underwent re-excision to obtain negative margins.

To identify variables associated with an increased LRR rate, we first performed Kaplan–Meier curves. All events were measured from the date of histological diagnosis. The statistical significance between survival curves was determined by a log-rank test between two groups. Then, significant variables were included in a multivariate analysis using Cox proportional hazards. The median follow-up period for surviving patients was 72 months. All tests were two tailed, and *p* < 0.05 was significant.

The prognostic index was developed by assigning a score of 0 (favourable) or a score of 1 (unfavourable) for each significant variable of multivariate analysis and was created separately for patients with BCS and mastectomy. The objective of the score was to create statistically different subgroups based on risk of LRR using the predictors. Because 26 patients had incomplete values for all variables, the data from 730 patients were used to perform our final analysis.

## Results

### Characteristics of the patients

A total number of 730 patients treated with NAC (327 patients treated with BCS and 403 patients treated with mastectomy) were included in the study.

Table 1 describes the distribution of patient and tumour characteristics of the study population. The median age of the patients treated with BCS was 49 ± 11, while 50 ± 10 of patients treated with mastectomy (*p* = 0.19). There was no difference between the distribution of patients younger than 40 years, 7% and 11% from breast-conservative surgery group and mastectomy group, respectively (*p* = 0.345).

Nearly, all of patients (97%) had stage II and III disease and only 3% of BCS has stage I, meanwhile 48% had stage II and 52% had stage III (*p* = 0.01).

The NAC regimen were generally doxorubicin and taxane based, with 63 % in BCS and 64% in mastectomy (*p* = 0.56) and chemotherapy with trastuzumab in 17% in BCS and 18% in mastectomy (*p* = 0.57). The NAC schedules followed the outlines of established protocols that were open during the study period in our centre.

In BCS group of patients, 24% patients underwent re-excision to obtain negative margins in 98% of patients.

All patients treated with BCS were treated with adjuvant external-beam radiotherapy to the breast with tangential fields. The median breast dose was 50 Gray (Gy) delivered in 25 fractions over 5 weeks, with 70% of patients receiving a tumour bed boost (median dose 10 Gy) using electrons. Radiotherapy to regional lymph nodes was delivered at supraclavicular fossa in 55% and at internal mammary areas in 12% in BCS group. While patients with mastectomy were treated with adjuvant external-beam radiotherapy with tangential fields to the chest wall in 82%, internal mammary area 32% and supraclavicular fossa 53%. All patients received the entire planned course of radiotherapy.

Adjuvant treatment was administered to 59% in the BCS group with hormone therapy versus 68% in the mastectomy group (*p* = 0.008). Meanwhile, 19% of BCS group received adjuvant chemotherapy versus 18% in the mastectomy group (*p* = 0.440)

Full details concerning treatment have been documented in Table 1.

### Locoregional recurrence score

At a median follow-up of 72 months, the 6-year cumulative incidence of LRR was 7.2% ( ± 3%) for BCS and 7.9% ( ± 3%) for mastectomy.

By univariate analysis, variables associated with an increased LRR were for BCS: HER2 positive, grade III, DCIS, No-pCR (ypTis, ypN0), and age < 40 years and for mastectomy: HER2 positive, DCIS, No-pCR, and LVI.

By multivariate analysis, variables associated with an increased LRR were for BCS: HER2 positive (HR: 11.1, *p* = 0.001), DCIS (HR: 3.1, *p* = 0. 005), and age < 40 years (HR: 2.8, *p* = 0.02) and for mastectomy: HER2 positive (HR: 9.5, *p* = 0.03), DCIS (HR: 2.7, *p* = 0.01), No-pCR (HR: 11.4, *p* = 0.01), and age < 40 years (HR: 2.8, *p* = 0.006).

Univariate and multivariate analyses details are described in Table 2.

The score for BCS is based in three factors (HER2 positive, DCIS, and age < 40 years) which correlated with LRR in univariate and multivariate analyses. Meanwhile, mastectomy is based on four factors (HER2 positive, DCIS, No-pCR, and age < 40 years).

The score stratified patients into three subsets with statistically different level of risk for LRR. For BCS, the 6-year LRR rates were 3%, 13%, and 33% for the low (score 0, *n* = 120), intermediate (score 1, *n* = 95), and high (score 2–3, *n* = 27) risk groups, respectively (*p* = 0.001). For mastectomy, the 6-year LRR rates were 0%, 8%, and 27% for the low (score 0, *n* = 20), intermediate (score 1–2, *n* = 191), and high (score 3–4, *n* = 30) risk groups, respectively (*p* = 0.001). Of note, 21 patients that had a LRR event were HER2 positive, of all of them had received trastuzumab.

**Figure 1.**
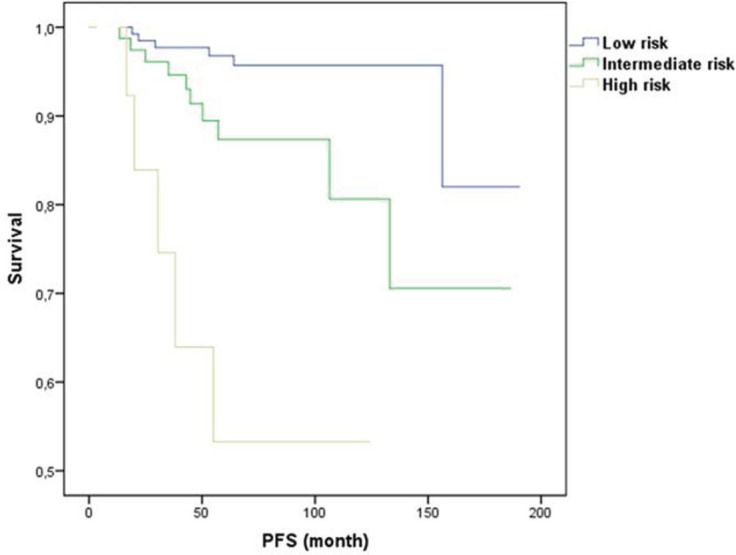
Prognostic index for LRR for conservative surgery.

**Figure 2.**
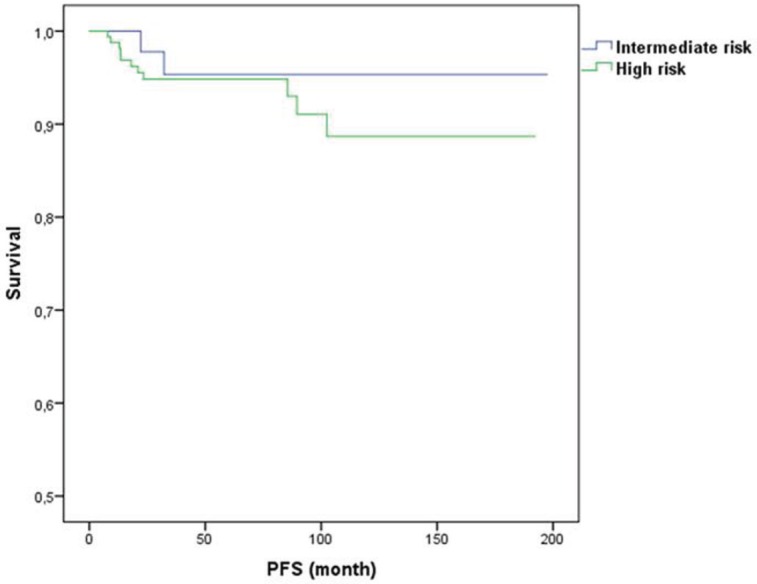
Prognostic index for LRR for mastectomy.

Figures 1 and 2 illustrate LRR survival for these three groups according to the prognostic index score for low, intermediate, and high risk in BCS group and mastectomy group.

## Discussion

The study identified specific subgroups at risk of LRR, among patients with breast carcinoma treated by BCT or mastectomy after NAC. Using the score, patients can be stratified into low-, intermediate-, or high-risk group for LRR depending on the kind of surgery that the patients underwent.

This prognostic index assumed some predefined criteria for the score validity. All patients treated with BCS were treated with adjuvant radiotherapy. Whole breast radiotherapy reduces the risk of local recurrence by two-thirds and it is associated with a survival benefit. Moreover, boost irradiation gives further 50% risk reduction and is indicated for patients with unfavourable risk factors for local control (age < 50 years, grade III tumour, vascular invasion, and non-radical tumour excision).

In contrast, post-mastectomy radiotherapy is recommended for women with positive nodes and for those T3–T4 tumours independent of nodal status [20, 21]. The role of regional radiotherapy has not been determined; it is indicated for patients with involved lymph nodes undergoing breast cancer or chest wall radiotherapy and should be considered for patients with pN0 and less than 10 nodes removed by axillary lymph node dissection, especially when other risk factors are also present. After axillary lymph node dissection, the resected part of the axilla should not be irradiated, except in cases of residual disease after surgery.

We would like to underline a study of patients treated with BCS after NAC at the University of Texas, M. D. Anderson Cancer Centre; they identified four risk factors that predicted LRR; clinical advanced lymph node disease (N2–3), pathological tumour size > 2 cm, multifocal residual disease and lymphovascular space invasion [20]. They developed the M. D. Anderson prognostic index (MDAPI) based on these four factors and stratified 340 patients into three subsets with statistically different levels of risk for LRR. Five-year free survival rates were 97%, 88%, and 82% for patients in the low-, intermediate- and high-risk group, respectively [22]. However, they conclude that further data are needed to define the risk of LRR after mastectomy to patients considered to be a high risk by MDAPI.

Another study reviews 3088 patients of two National Surgical Adjuvant Breast and Bowel Project (NSABP) neoadjuvant trials, and the 10-year cumulative incidence of LRR was 12.3% for mastectomy patients and 10.3% for BCS. The study concluded that independent predictors of LRR in patients with BCS were age, clinical nodal status, and pathological nodal status/breast tumour response; in mastectomy patients, they were clinical tumour size, clinical nodal status, and pathological nodal status/breast tumour response [23].

In this study, there are no differences between BCS or mastectomy because, at a median follow-up of 72 months, the 6-year cumulative incidence of LRR was 7.2% (±3%) for BCS and 7.9% (±3%) for mastectomy. The reason behind could be that patients who underwent a mastectomy had 52% of stage III versus 17% in BCS group.

The score for BCS is based in three factors (HER2 positive, DCIS, and age < 40 years) which correlated with LRR in univariate and multivariate analyses. Meanwhile, mastectomy is based on four factors (HER2 positive, DCIS, No-pCR, and age < 40 years).

The score enabled the identification of a small cohort of patients with high risk of developing LRR after NAC followed by BCS or mastectomy. Specifically, for patients treated with BCS with score 2 or 3(HER2-positive, DCIS, and age < 40 years), a 6-year LRR rate of 33% was observed. And patients that underwent a mastectomy with score 3 or 4 (HER2 positive, DCIS, No-pCR, and age < 40 years) had a 6-year LRR rate of 27%. This subgroup of patients represent only 7% of the population, but they are young women (< 40 years, Her2 positive, and without pCR). While our findings may not change current surgery options, this high-risk subgroup perhaps may benefit from alternative strategies of treatment such as immunological therapies that may be able to decrease this risk of LRR.

Recent studies suggest that tumour-infiltrating lymphocytes (TILs) are associated with DFS in operable Her2-overexpressiong breast cancer. Further research in TILs could be useful as stratification to guide immunological therapeutic approaches [26].

In contrast, the score also identified a favourable subgroup of patients (with 1 for BCS and 1 or 2 score for mastectomy) who had 6-year LRR rates of 3% and 0%, respectively. This subgroup only represented 19% of the study population.

The results of this study should be interpreted in the context of its potential weaknesses. First, patients included in the study were treated from 1994 to 2014, and this is especially relevant given that neoadjuvant anti-HER2 therapy was not available until 2011 [24, 25].

However, of 21 patients that had a LRR event who were HER2 positive, all of them had received trastuzumab.

## Conclusion

In conclusion, the score is a tool that could be useful in order to predict much better the risk of LRR after BCS and mastectomy. By clinicopathological findings, patients can be stratified into three different prognostic groups. Patients with a score of 0–1, which made up to 19% of the study population, had very low rates of LRR. The score enabled the identification of a small group (7%) of patients with very high risk of LRR and who may benefit from alternative or additional locoregional treatment strategies. However, further prospective data are needed to validate this score in an independent data set.

## Figures and Tables

**Table 1.** Patientsʼ characteristics according to type of surgery (%).

Characteristic	Type of Surgery		Chi-Square
	Conservative	Mastectomy	
**Age <40 years**	7	11	p.345
**Clinical TNM stage**			
Stage I	3	0	p.001
Stage II	80	48	
Stage III	17	52	
**Histology**			
Ductal	90	78	p.002
Lobular	10	22	
**ER-Positive/HER2-negative**	59	66	p.200
**HER2-Positive**	27	21	
**Triple Negative**	14	13	
**Ki67>20**	12	13	p.242
**Type of NAC**			
Anthacyclines plus taxanes	64	63	p.570
Trastuzumab based	18	17	p.571
**Pathologic Complete Response** (ypT0/is, ypN0)	13	10	p.001
**Residual Tumor Morphology**			
Unifocal Disease	24	33	p.051
Multifocal Disease	9	13	
Unknown	54	44	
**Extensive DCIS in specimen**	7	11	p.001
**Re-Excision**	24	0	p.002
**Negative Margins**	98	99	p.002
**Radiation to:**			
Breast	98	0	p.001
Chest Wall	0	82	p.002
Internal Mammary	12	32	p.002
Supraclavicular Fose	55	53	p.273
**Radiotherapy Boost**			
Yes	77	52	p.001
External	43	100	
Interstitial	57	0	
**Adjuvant Hormonotherapy**	59	68	p.008
**Adjuvant Trastuzumab**	19	18	p.440

**Table 2.** Univariate analysis for 6 year LRR rate.

Conservative surgery			
Variable	6 year LRR rate	P (log-rank test)	
**ER/HER2 status**			
ER-positive/HER2-negative	3	p.002	
Triple Negative	6		
HER2-positive	20		
**Grado**			
Grado I	4	p.020	
Grado II	7		
Grado III	11		
**CDIS**			
Yes	20	p.001	
No	2		
**pCR (ypTis(ypN0)**			
Yes	2	p.030	
No	20		
**Age, years**			
** <40**	14	p.001	
** >40**	6		
**Mastectomy**			
Variable	6 year LRR rate	P (log-rank test)	
**ER/HER2 status**			
ER-positive/HER2-negative	2	p.001	
Triple Negative	11		
HER2-positive	17		
**CDIS**			
Yes	17	p.001	
No	6		
**pCR (ypTis(ypN0)**			
Yes	2	p.030	
No	16		
**Age, years**			
<40	16	p.001	
>40	6		
**Lymphovascular Invasion**			
Yes	13	p.010	
No	6		
